# Perceived similarity determines social comparison effects of more and less physically active others

**DOI:** 10.1177/13591053221086759

**Published:** 2022-04-18

**Authors:** Iris Perey, Joerg Koenigstorfer

**Affiliations:** Technical University of Munich, Munich, Germany

**Keywords:** exercise, physical activity, self-efficacy, self-evaluation, social comparison

## Abstract

This research tested whether the effects of physical activity (PA) comparisons depend on the perceived similarity to comparison standards. In 3 experimental studies, participants compared themselves to a more or a less physically active person. Results showed that perceived similarity determined comparison outcomes: Participants’ PA self-evaluation and self-efficacy were higher when focusing on similarities with more (vs less) (Study 1) and dissimilarities with less (vs more) active others (Study 1 and 2). Considering the opposite of the impression that less active others are similar and more active others are dissimilar increased participants’ PA self-evaluation, self-efficacy, and intention (Study 3).

## Introduction

Despite evidence that regular physical activity (PA) results in mental and physical health benefits (Warburton and Bredin, 2017), more than a quarter of the global adult population do not meet the World Health Organization’s recommendations for sufficient PA (Guthold et al., 2018). Accordingly, the promotion of PA presents an urgent target within health psychology. In line with theoretical frameworks (Bandura, 2004; Ryan and Deci, 2007), social influences contribute to whether and to what extent individuals engage in PA (Bauman et al., 2012). One particular social influence that predicts PA is the comparison with others (Diel and Hofmann, 2019). However, the factors determining whether a comparison results in beneficial (i.e. health-promoting) or adverse outcomes, as well as the underlying mechanisms explaining these effects, remain largely undiscovered. In addition, research on strategies that could be applied to modify PA comparison consequences is lacking. These investigations are likely to yield useful implications for the effective use of social comparison in the promotion of PA.

### Social comparison and PA self-evaluation and self-efficacy

Comparisons with others are a ubiquitous part of everyday life that shapes how we think about our abilities and performances (Marsh et al., 2020). In the realm of PA, social comparisons may play a crucial role in the formation of two specific types of self-beliefs, namely *PA self-evaluation* and *self-efficacy*. First, the evaluation of one’s PA is predicted by comparisons in this context. For example, Diel et al. (2021) showed that athletes evaluated their performance improvement more positively when they engaged in upward comparison, but more negatively when they engaged in downward comparison. Second, self-efficacy, the belief in one’s capability to execute a behavior, increases when observing other people mastering comparable activities (i.e. vicarious experience; Bandura, 1977). For instance, seeing a friend finishing a marathon may raise the belief that one can also do so. As social comparison offers insights about others’ successes and failures, it is considered a specific type of vicarious experience that impacts efficacy beliefs (Carmona et al., 2008).

### The moderating role of perceived similarity

Social comparisons can have positive and negative impacts on the comparer. While comparisons with others performing better (i.e. *upward comparisons*) can inspire self-improvement, they can also be discouraging (Bellizzi et al., 2006; Corcoran et al., 2011). Comparisons with others performing worse (i.e. *downward comparisons*) can boost self-views, but can also lead to reduced efforts to improve and pursue goals (Corcoran et al., 2011; Diel and Hofmann, 2019). A comparison’s outcome can thus not be determined by the *comparison direction* alone, suggesting that additional factors are involved.

One such factor may be the *perceived similarity* to the comparison standard, which can be understood through the lens of the selective accessibility model (Mussweiler, 2003): When faced with a comparison opportunity, individuals first make a rapid judgment of overall similarity between themselves and the standard and subsequently focus on information consistent with this initial assessment. As a result, focusing on information confirming similarity is likely to lead to assimilation (i.e. moving self-beliefs toward the standard) and focusing on information confirming dissimilarity is likely to lead to contrast (i.e. moving self-beliefs away from the standard). Taken together, comparisons should have positive impacts when focusing on similarities with upward and dissimilarities with downward standards (Mussweiler, 2003).

Preliminary evidence supports the role of perceived similarity in PA comparisons. Diel and Hofmann (2019) manipulated standard extremity (moderate vs extreme), assuming that similarity is high for moderate and low for extreme standards. They found that comparison consequences did depend on the standard extremity: Moderate upward standards motivated participants to exercise, moderate downward and extreme upward standards held less but some motivational potential, and extreme downward standards were demotivating. Nevertheless, perceived similarity was manipulated indirectly and its potential moderating effect in the context of PA comparisons remains largely unexplored. We suggest that perceived similarity will moderate the effect of comparison direction on PA self-evaluation and self-efficacy. We predict that individuals will evaluate their PA more positively and feel more efficacious to engage in PA if they focus on similarities with an upward (vs downward) standard. Conversely, individuals should have higher PA self-evaluation and self-efficacy levels if they focus on dissimilarities with a downward (vs upward) standard.

### The mediating roles of self-evaluation and self-efficacy

Self-evaluation and self-efficacy present important sources for goal commitment and self-improvement (Garcia et al., 2013; Martin et al., 2016). Such self-beliefs are central to health behavior change theories (Bandura, 2004; Ryan and Deci, 2007) and empirical evidence supports their role in the formation of health behaviors like PA. Individuals high in self-efficacy were, for example, found to be more likely to be physically active than those low in self-efficacy (Bauman et al., 2012). Hence, comparison effects on PA self-evaluation and self-efficacy may be positively associated with PA intention. As both self-beliefs mediate the links between social environmental factors and PA intention and behavior (Kim et al., 2021; Puente and Anshel, 2010), we suggest that they will mediate the link between comparison direction and PA intention.

### Considering the opposite as a strategy for influencing PA comparison effects

Given that comparisons with better and worse performing others have benefits but also drawbacks, it is vital to explore strategies that reduce negative outcomes and promote PA. One approach may be the *consider-the-opposite strategy* (Lord et al., 1984), which involves generating evidence contradicting one’s initial beliefs. By increasing the accessibility of previously neglected information, considering the opposite reduces people’s tendency to rely on initial beliefs when making judgments (Mussweiler et al., 2000).

There is currently no evidence for the application of the consider-the-opposite strategy in the realm of social comparison. As perceived similarity to a standard presents a specific initial belief that may determine the focus of subsequent information processing, we expect that the consider-the-opposite strategy can be used to elicit information contrary to the initially perceived similarity and ultimately alter comparison outcomes. Specifically, we propose that PA self-evaluation, self-efficacy, and intention will increase after participants consider the opposite of their initial focus on dissimilarities with an upward and similarities with a downward standard. For example, someone who originally believes to be different from an active person should recognize that the two of them have more in common when consciously thinking about similarities. This should improve the way the comparer evaluates, feels efficacious about, and intends to engage in PA. We expect a decrease in these values after participants consider the opposite of their initial focus on similarities with an upward and dissimilarities with a downward standard. Noticing, for instance, that the own performance deviates more from that of an active person than initially assumed should diminish one’s PA self-evaluation, self-efficacy, and intention.

### The present research

The first aim of the present research is to test the proposed moderated mediation model, including comparison direction (predictor), perceived similarity (moderator), and PA self-evaluation and self-efficacy (mediators) for the prediction of PA intention (outcome). The second aim of this research is to explore whether comparison effects can be influenced via the consider-the-opposite strategy.

## Study 1

In Study 1, we manipulated the comparison direction (upward vs downward) and subsequently assessed participants’ perceived similarity to the standard, PA self-evaluation, self-efficacy, and intention.

### Methods

#### Participants and procedure

Participants were recruited through Amazon’s Mechanical Turk (MTurk). MTurk workers from the U.S. between 18 to 64 years old were eligible for participation. A G*Power analysis (Faul et al., 2007) indicated a required sample size of *N* = 92, assuming a medium effect size (*f*^2^ = 0.15), α = 0.05, power = 0.80, and a multiple linear regression model with 5 predictors. The sample included 240 respondents (Table 1). Participants received US-$1.50 as compensation. All study procedures followed the ethical principles of the Declaration of Helsinki (World Medical Association, 2013).

**Table 1.** Participants’ demographic characteristics and past-week physical activity for Study 1, 2, and 3.

	Study 1		Study 2		Study 3	
	*M* (SD)	Range	*M* (SD)	Range	*M* (SD)	Range
Age	41.00 (11.05)	18.00–64.00	40.56 (10.72)	20.00–64.00	40.96 (10.76)^a^	20.00–64.00
BMI	26.60 (7.39)^a^	14.06–58.82	26.70 (5.82)	16.80–47.76	27.02 (5.94)	14.43–52.12
Past-Week PA (MET minutes)	2709 (2766)	0–13,968	2895 (3676)^a^	0–23,172	2899 (3501)	0–25,998
	*n*	%	*n*	%	*n*	%
Gender						
Female	129	53.75	147	59.27	131	53.69
Male	111	46.25	98	39.52	108	44.26
Non-binary	0	0.00	2	0.81	2	0.82
Other	0	0.00	0	0.00	1	0.41
Prefer not to say	0	0.00	1	0.40	2	0.82
Education level						
High school degree or less	29	12.08	35	14.11	20	8.20
Some college	76	31.67	56	22.58	75	30.74
Bachelor’s degree	99	41.25	119	47.98	112	45.90
Master’s degree	31	12.92	35	14.11	32	13.11
Doctorate	5	2.08	1	0.40	3	1.23
Prefer not to say	0	0.00	2	0.81	2	0.82
Ethnic background						
White/Caucasian	194	80.83	202	81.45	192	78.69
Hispanic/Latino	14	5.83	12	4.84	5	2.05
Black/African American	15	6.25	15	6.05	19	7.79
Asian	11	4.58	12	4.84	22	9.02
Other	5	2.08	5	2.02	2	0.82
Prefer not to say	1	0.42	2	0.81	4	1.64

MET = metabolic equivalent of task; PA = physical activity.

a Conditions differed significantly. The pattern of the main analyses results did not change when condition differences were accounted for by including affected variables as covariates.

Participants first gave informed consent and indicated their gender to provide them with a same-sex standard description for the social comparison task.^1^ They were then asked to complete the social comparison task, which was described as a pretest of stimulus material for another study. Subsequently, participants completed the measures described below in the listed order. For PA-related measures, it was noted that PA refers to any bodily movement that requires energy expenditure.

#### Social comparison task

About half of the participants read the description of an upward standard (*upward condition*), while the other half read the description of a downward standard (*downward condition*; Supplemental Material). The upward standard was described as a person who exercises regularly, incorporates movement into everyday life, and has good physical abilities. The downward standard was described as a person who does not exercise, avoids movement in everyday life, and has poor physical abilities. Participants were instructed to write a few sentences about how their PA compares to the standard. For statistical analyses, the dummy-coded variable *comparison direction* (i.e. 0 = upward; 1 = downward) was created.

#### Measures

##### Perceived similarity

Similarity was measured by four items asking about the extent to which participants focused on similarities (e.g. “While comparing yourself to [*Name*], how much did you focus on similarities between yourself and [*Name*]?”; α = 0.96) and four items on the extent to which participants focused on dissimilarities with the standard (e.g. “While comparing yourself to [*Name*], how much did you focus on differences between yourself and [*Name*]?”; α = 0.97). Items were rated from 1 = *not at all* to 5 = *most of the time* (Arigo et al., 2015). Separate mean scores were calculated.^2^

##### PA self-evaluation

PA self-evaluation was assessed with three items (“How satisfied are you with your level of PA?”, “How physically active do you find yourself?”, “How good do you feel about your level of PA?”; α = 0.97) rated from 1 = *not at all* to 7 = *very much* (Papies and Nicolaije, 2012).

##### PA self-efficacy

PA self-efficacy was measured with three items (“I am confident that I can find means and ways to be physically active,” “I am confident that I can accomplish my PA goals that I set,” “I am confident that I can overcome barriers and challenges with regard to PA if I try hard enough”; α = 0.90) rated from 1 = *not at all true* to 4 = *always true* and drawn from the Exercise Self-Efficacy Scale (Kroll et al., 2007).

##### PA intention

Based on guidelines for measuring intention (Ajzen, 2019), three items (“I intend to be physically active over the next 7 days,” “I will frequently exercise in the upcoming 7 days,” “Next week, I will be physically active in my daily life”; α = 0.95) were rated from 1 = *strongly disagree* to 7 = *strongly agree*.

##### Past-week PA

For descriptive purposes, time spent being physically active over the past week was assessed using the International PA Questionnaire Short Form (IPAQ-SF; Craig et al., 2003). Guidelines for data processing (IPAQ Research Committee, 2005) were followed to calculate metabolic equivalent of task minutes.

##### Perceived comparison direction

To test whether the comparison direction manipulation was successful, one item (“Compared to [Name], I am . . .”), rated from 1 = *much less physically active* to 7 = *much more physically active*, was included.

##### Demographics

In addition to gender and age, participants indicated their ethnicity, education, height, and weight.

### Results

As evidence of a successful manipulation, participants in the upward condition rated themselves to be less physically active than the standard (*M* = 2.13, SD = 1.19), whereas the opposite was true in the downward condition (*M* = 5.79, SD = 1.13), *t*(238) = −24.46, *p* < 0.001.

We estimated a moderated mediation model using PROCESS Model 7 (Hayes, 2015) with 5000 bootstrap samples to test whether similarity moderated the potential indirect relationship between comparison direction and PA intention via self-evaluation and self-efficacy. To identify the nature of the interaction, conditional effects at low (mean −1 SD) and high (mean + 1 SD) similarity were inspected. Continuous variables were standardized prior to analyses.

**Figure 1.**
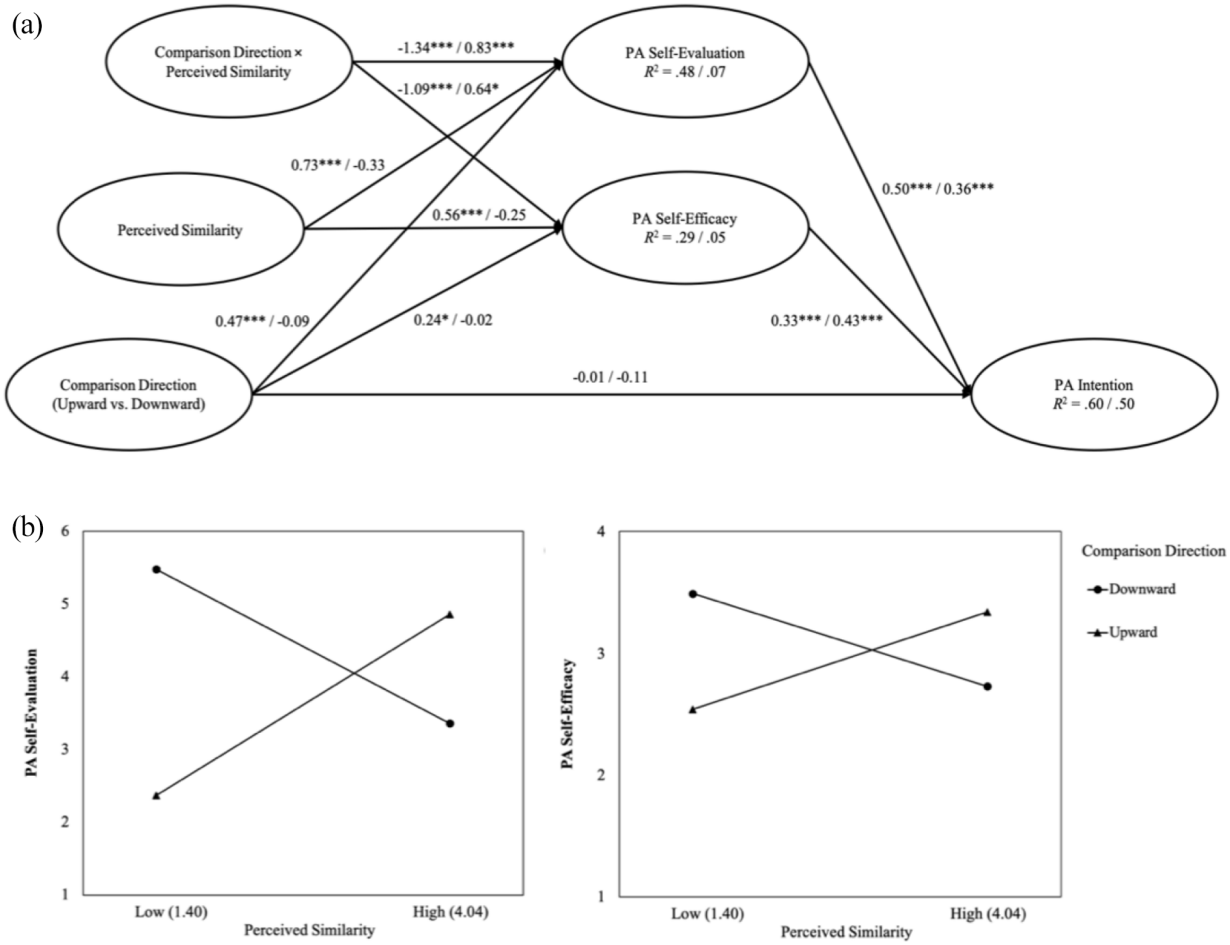
Moderated Mediation Results for Study 1 and Study 2 (a) with Conditional Effects of Comparison Direction on PA Self-Evaluation and Self-Efficacy at Low (Mean – 1 SD) and High (Mean + 1 SD) Perceived Similarity for Study 1 (b). Perceived similarity constitutes a continuous variable in Study 1 and a categorical variable (similarity vs dissimilarity) in Study 2. Coefficients to the left of the slash refer to Study 1 and the ones to the right of the slash refer to Study 2; PA = physical activity; response scales ranged from 1 = *not at all* to 7 = *very much* for PA self-evaluation, from 1 = *not at all* true to 4 = *always true* for PA self-efficacy, and from 1 = *not at all* to 5 = *most of the time* for perceived similarity; *p <0.05, *** *p* < 0.001 (two-tailed); coefficients are unstandardized.

Results revealed significant Comparison Direction × Similarity effects on PA self-evaluation, *B* = −1.63, SE = 0.10, *p* < 0.001, and self-efficacy, *B* = −1.09, SE = 0.11, *p* < 0.001 (Figure 1a). Conditional effects of comparison direction on PA self-evaluation were significant for low, *B* = 1.81, SE = 0.14, *p* < 0.001, and high similarity, *B* = −0.87, SE = 0.14, *p* < 0.001. Conditional effects of comparison direction on PA self-efficacy were significant for low, *B* = 1.33, SE = 0.16, *p* < 0.001, and high similarity, *B* = −0.84, SE = 0.16, *p* < 0.001. In other words, when similarity to the standard was high, participants comparing upward had higher PA self-evaluation and self-efficacy levels than those comparing downward. When similarity was low, PA self-evaluation and self-efficacy were greater for participants comparing downward (vs upward) (Table 2; Figure 1b).

**Table 2.** Means and standard deviations of PA self-evaluation and self-efficacy per condition for Study 1 and 2.

Measures	Study 1			Study 2		
		PA Self-evaluation	PA Self-efficacy		PA self-evaluation	PA self-efficacy
	*n*	*M* (SD)	*M* (SD)	*n*	*M* (SD)	*M* (SD)
Upward Similarity	120	4.86 (1.60)	3.35 (0.78)	58	4.03 (1.71)	3.10 (0.74)
Downward Similarity	120	3.35 (2.06)	2.74 (1.00)	65	3.87 (1.76)	3.09 (0.62)
Upward Dissimilarity	120	2.32 (2.00)	2.53 (0.97)	65	3.45 (1.74)	2.93 (0.69)
Downward Dissimilarity	120	5.47 (1.63)	3.49 (0.79)	60	4.73 (1.59)	3.36 (0.57)

PA = physical activity. Response scales ranged from 1 = *not at all* to 7 = *very much* for PA self-evaluation and from 1 = *not at all true* to 4 = *always true* for PA self-efficacy. For Study 1, scores at low (mean – 1 SD) and high (mean + 1 SD) perceived similarity are presented.

Tests of conditional indirect effects suggest that the relationship between comparison direction and PA intention was mediated by PA self-evaluation at low, *B* = 0.90, SE = 0.15, 95% CI [0.61; 1.19], and high similarity, *B* = −0.43, SE = 0.10, 95% CI [−0.63; −0.24]. The relationship between comparison direction and PA intention was mediated by PA self-efficacy at low, *B* = 0.45, SE = 0.13, 95% CI [0.23; 0.71], and high similarity, *B* = −0.29, SE = 0.10, 95% CI [−0.52; −0.12]. The results further revealed significant moderated mediation indices for PA self-evaluation, *B* = −0.66, SE = 0.11, 95% CI [−0.88; −0.44] and self-efficacy, *B* = −0.37, SE = 0.11, 95% CI [−0.59; −0.18].

### Discussion

Study 1 showed that the effects of upward and downward comparisons depend on the extent to which similarities and differences between the self and the standard are recognized. Participants who focused on similarities with the standard evaluated their PA more positively and felt more efficacious to engage in PA if they read about an upward standard. Participants who focused on dissimilarities had higher levels of PA self-evaluation and self-efficacy if they read about a downward standard. Further, PA self-evaluation and self-efficacy explained the association between comparison direction and intention. These findings provide a potential explanation for the differential effects observed in research examining PA upward and downward comparisons in isolation (Lockwood et al., 2005) and add to investigations of factors moderating PA comparison effects (e.g. standard extremity; Diel and Hofmann, 2019).

While perceived similarity was measured in Study 1, Study 2 tested whether experimentally manipulating perceived similarity had similar effects. These insights would offer implications regarding how PA comparisons could be influenced so that positive self-beliefs and the willingness to be active are promoted.

## Study 2

Study 2 aimed to replicate the results of Study 1, using a similarity manipulation (similarity vs dissimilarity) first and a comparison direction manipulation (upward vs downward) second.

### Methods

#### Participants and procedure

The recruitment and procedures for Study 2 resembled those of Study 1. In total, 248 participants took part in the study (Table 1). A similarity manipulation was added to the comparison task: Before reading the upward or downward standard description, participants were instructed to either focus on similarities or differences in terms of PA. Accordingly, participants were randomly assigned to one of the following conditions: *upward similarity, downward similarity, upward dissimilarity*, and *downward dissimilarity*. Similarity items were used as manipulation checks and assessed as the last measure before the demographics.

#### Measures

The measures were identical to those used in Study 1. Cronbach’s α was 0.94, 0.87, 0.94, and 0.96 for PA self-evaluation, self-efficacy, intention, similarity, respectively.

### Results

Participants in the upward conditions (*M* = 1.95, SD = 1.17) judged themselves to be less physically active than the standard, while participants in the downward conditions (*M* = 5.81, SD = 1.17) judged themselves to be more physically active than the standard, *t*(246) = −25.94, *p* < 0.001. Participants in the similarity conditions indicated a greater similarity to the standard (*M* = 3.60, SD = 1.30) than those in the dissimilarity conditions (*M* = 2.49, SD = 1.34), *t*(246) = 6.65, *p* < 0.001.

Analyses identical to those applied in Study 1 were used to test for moderated mediation. As expected, the Comparison Direction × Similarity effects on PA self-evaluation, *B* = 0.83, SE = 0.25, *p* < 0.001, and self-efficacy, *B* = 0.64, SE = 0.25, *p* = 0.01, were significant (Figure 1a). Conditional effects of comparison direction on PA self-evaluation were significant for the dissimilarity, *B* = 0.73, SE = 0.17, *p* < 0.001, and unexpectedly, non-significant for the similarity condition, *B* = −0.09, SE = 0.18, *p* = 0.60. Conditional effects of comparison direction on PA self-efficacy were significant for the dissimilarity, *B* = 0.62, SE = 0.18, *p* < 0.001, but non-significant for the similarity condition, *B* = −0.02, SE = 0.18, *p* = 0.93. Participants focusing on differences with a downward standard evaluated their PA more positively and felt more efficacious to be active than participants focusing on differences with an upward standard (Table 2).

Conditional indirect effects of comparison direction on PA intention via PA self-evaluation were significant for the dissimilarity, *B* = 0.27, SE = 0.08, 95% CI [0.13; 0.44], and non-significant for the similarity condition, *B* = −0.03, SE = 0.07, 95% CI [−0.18; 0.10]. Conditional indirect effects of comparison direction on PA intention via PA self-efficacy were significant for the dissimilarity, *B* = 0.27, SE = 0.08, 95% CI [0.12; 0.44], but non-significant for the similarity condition, *B* = −0.01, SE = 0.08, 95% CI [−0.16; 0.16]. Indices of moderated mediation were significant for PA self-evaluation, *B* = 0.30, SE = 0.11, 95% CI [0.11; 0.53], and self-efficacy, *B* = 0.28, SE = 0.11, 95% CI [0.06; 0.50].

### Discussion

Study 2 demonstrated that participants had higher PA self-evaluation and self-efficacy levels when they focused on dissimilarities with a downward standard. Surprisingly, participants who focused on similarities with an upward standard did not exhibit greater PA self-evaluation and self-efficacy. This finding may be explained in light of recent meta-analysis results (Gerber et al., 2018), which suggest that individuals typically move their self-beliefs away from, rather than toward, a standard when making comparisons. The authors conclude that a focus on similarities is harder to induce than a focus on dissimilarities and may require priming rather than explicit induction. Nevertheless, the current findings yield experimental evidence for the crucial role of perceived similarity in PA comparisons.

The findings show that the perception of dissimilarity can be induced by explicit instruction to seek differences with others *before* overall similarity is assessed, and doing so has important downstream effects. It is, however, unclear whether there are similar effects when the perception of similarity or dissimilarity is manipulated *after* the initial judgment of (dis)similarity has already taken place. This drawback was addressed by Study 3, in which we examined the consider-the-opposite strategy as a potential debiasing strategy for modifying comparison consequences.

## Study 3

Study 3 aimed to test whether considering the opposite of one’s initial similarity judgment would impact comparison outcomes. We first manipulated the comparison direction (upward vs downward) and assessed similarity and outcome measures. Second, we employed the consider-the-opposite strategy and measured outcomes again.

### Methods

#### Participants and procedure

A G*Power analysis (Faul et al., 2007) predicted a required sample size of *N* = 92, assuming a medium effect size, α = 0.05, power = 0.80, and a repeated measures design with four groups and two measurements. A total of 244 respondents took part in the study (Table 1). In the first part (T1), participants engaged in the social comparison task used in Study 1, indicated whether they focused on similarities or differences, and completed outcome measures. In the second part (T2), they engaged in a second comparison with the same standard but were asked to consider the opposite of their initially reported similarity and to list as many similarities (vs differences) between the self and the standard, in terms of PA, as they could find. Subsequently, participants completed outcome measures again.

Participants were randomly assigned to one of the following conditions: upward similarity at T1 with a dissimilarity focus using the consider-the-opposite strategy at T2 (*upward initial similarity consider-the-opposite*), upward dissimilarity at T1 with a similarity focus using the consider-the-opposite strategy at T2 (*upward initial dissimilarity consider-the-opposite)*, downward similarity at T1 with a dissimilarity focus using the consider-the-opposite strategy at T2 (*downward initial similarity consider-the-opposite*), downward dissimilarity at T1 with a similarity focus using the consider-the-opposite strategy at T2 (*downward initial dissimilarity consider-the-opposite)*. To reinforce participants’ willingness to think about information contradicting their initial impression, it was added to the task description that, during comparisons, people tend to notice similarities (vs differences) at first but become aware of differences (vs similarities) when taking a closer look. Similarity items were used as manipulation checks.

To provide a rationale for the use of the consider-the-opposite strategy, participants were told that the study would aim to explore people’s ability to adapt their thinking. One filler item (i.e. “Overall, how difficult or easy did you find the task to adapt your thinking?”), rated from 1 = *very difficult* to 5 = *very easy*, was used to substantiate the cover story. Participants received US-$2.00 as compensation. The remainder of the procedure was identical to Study 1.

#### Measures

*Consider-the-opposite*. The dummy-coded variable consider-the-opposite (i.e. 0 = initial similarity; 1 = initial dissimilarity) was created. We used one item to assess perceived similarity at T1 (i.e. “While comparing yourself to [*Name*], did you rather focus on similarities or differences between yourself and [Name]?”), which was answered with “*I rather focused on similarities between myself and [*Name*]*” or “*I rather focused on differences between myself and [Name]*.”

The remaining measures resemble those of Study 1. Cronbach’s α was 0.98 for similarity, 0.96, 0.90, and 0.95 for PA self-evaluation, self-efficacy, and intention at T1, respectively, and 0.97, 0.92, and 0.96 at T2.

### Results

As anticipated, participants in the upward conditions judged themselves to be less active than the standard (*M* = 2.40, SD = 1.43), while participants in the downward conditions judged themselves to be more active than the standard (*M* = 5.65, SD = 1.26), *t*(242) = −18.84, *p* < 0.001. Further, participants focusing on similarities during the consider-the-opposite task indicated greater similarity to the standard (*M* = 4.30, SD = 0.80) than those focusing on differences (*M* = 2.34, SD = 1.31), *t*(242) = −14.50, *p* < 0.001.

Separate mixed ANOVAs were conducted to test the potential three-way interaction between Comparison Direction (upward vs downward), Consider-The-Opposite (initial similarity vs initial dissimilarity), and Time (T1 vs T2) on PA self-evaluation, self-efficacy, and intention. Simple effects of Time within Comparison Direction and Consider-The-Opposite were inspected to identify for which conditions the time differences existed.

Results revealed a significant Time × Comparison Direction × Consider-The-Opposite effect on PA self-evaluation, *F*(1, 240) = 30.30, *p* < 0.001, $\eta_{p}^{2}$ =0.11 (Table 3). Self-evaluation increased from T1 to T2 in the upward initial dissimilarity, *F*(1, 240) = 28.29, *p* < 0.001, $\eta_{p}^{2}$ =0.11, and the downward initial similarity consider-the-opposite conditions, *F*(1, 240) = 6.82, *p* = 0.01, $\eta_{p}^{2}$ =0.03. There was a marginally significant decrease in self-evaluation in the downward initial similarity consider-the-opposite condition, *F*(1, 240) = 3.18, *p* = 0.08, $\eta_{p}^{2}$ =0.03, and no significant difference in self-evaluation in the upward initial similarity consider-the-opposite condition, *F*(1, 240) = 2.64, *p* = 0.11, $\eta_{p}^{2}$ =0.01.

**Table 3.** Condition differences in study variables over time for Study 3.

Condition	Constructs								
	PA self-evaluation			PA self-efficacy			PA intention		
	T1	T2	Simple effects of time	T1	T2	Simple effects of time	T1	T2	Simple effects of time
	*M* (SD)	*M* (SD)	$\eta_{p}^{2}$ ; *F* tests	*M* (SD)	*M* (SD)	$\eta_{p}^{2}$ ; *F* tests	*M* (SD)	*M* (SD)	$\eta_{p}^{2}$ ; *F* tests
Upward initial similarity consider-the-opposite (n = 47)	5.49 (1.32)	5.30 (1.47)	0.01; 2.64	3.51 (0.50)	3.42 (0.51)	0.01; 2.28	6.17 (1.00)	6.20 (0.93)	0.00; 0.08
Downward initial similarity consider-the-opposite (n = 38)	2.75 (1.60)	3.10 (1.76)	0.03; 6.82**	2.63 (0.71)	2.78 (0.84)	0.02; 4.83*	3.98 (1.73)	4.36 (1.95)	0.05; 12.36***
Upward initial dissimilarity consider-the-opposite (n = 69)	2.72 (1.69)	3.24 (1.80)	0.11; 28.29***	2.79 (0.75)	3.03 (0.80)	0.08; 22.08***	4.20 (1.85)	4.59 (1.91)	0.09; 23.06***
Downward initial dissimilarity consider-the-opposite (n = 90)	4.75 (1.46)	4.60 (1.66)	0.01; 3.18	3.34 (0.61)	3.24 (0.70)	0.02; 5.53*	5.79 (1.41)	5.78 (1.38)	0.00; 0.003
	$\eta_{p}^{2}$ ; *F* test			$\eta_{p}^{2}$ ; *F* test			$\eta_{p}^{2}$ ; *F* test		
Time × Comparison Direction × Consider-The-Opposite	0.11; 30.30***			0.10; 26.40***			0.07; 16.89***		

Simple effects of Time within Comparison Direction and Consider-The-Opposite; PA = physical activity; response scales ranged from 1 = *not at all* to 7 = *very much* for PA self-evaluation, from 1 = *not at all true* to 4 = *always true* for PA self-efficacy, and from 1 = *strongly disagree* to 7 = *strongly agree* for PA intention; *p <0.05, ** *p* < 0.01, *** *p* < 0.001 (two-tailed).

The Time × Comparison Direction × Consider-The-Opposite effect on PA self-efficacy was significant, *F*(1, 240) = 26.40, *p* < 0.001, $\eta_{p}^{2}$ =0.10. Self-efficacy increased from T1 to T2 in the upward initial dissimilarity, *F*(1, 240) = 22.08, *p* < 0.001, $\eta_{p}^{2}$ =0.08, and the downward initial similarity consider-the-opposite conditions, *F*(1, 240) = 4.83, *p* = 0.03, $\eta_{p}^{2}$ =0.02. Self-efficacy decreased in the downward initial dissimilarity consider-the-opposite condition, *F*(1, 240) = 5.53, *p* = 0.02, $\eta_{p}^{2}$ =0.02. In the upward initial similarity consider-the-opposite condition, there was no change in self-efficacy, *F*(1, 240) = 2.28, *p* = 0.13, $\eta_{p}^{2}$ =0.01.

The Time × Comparison Direction × Consider-The-Opposite effect on PA intention was significant, *F*(1, 240) = 16.89, *p* < 0.001, $\eta_{p}^{2}$ =0.07. Intention increased from T1 to T2 in the upward initial dissimilarity, *F*(1, 240) = 23.06, *p* < 0.001, $\eta_{p}^{2}$ =0.09, and the downward initial similarity consider-the-opposite conditions, *F*(1, 240) = 12.36, *p* = 0.001, $\eta_{p}^{2}$ =0.05. There were no significant changes in intention in both the downward initial dissimilarity and the upward initial similarity consider-the-opposite conditions, *F*(1, 240) = 0.003, *p* = 0.96, $\eta_{p}^{2}$ =0.00 and *F*(1, 240) = 0.08, *p* = 0.77, $\eta_{p}^{2}$ =0.00, respectively.^3^

### Discussion

Study 3 showed that PA self-evaluation, self-efficacy, and intention increased from T1 to T2 if participants considered the opposite of their initial focus on dissimilarities with an upward and similarities with a downward standard. These findings offer preliminary evidence for the consider-the-opposite strategy as an effective tool for debiasing similarity perceptions, which may bring about improvements in how individuals evaluate their PA as well as how capable they feel and how willing they are to execute PA.

## General discussion

In 3 studies, we have shed light on how social comparisons impact PA self-evaluation, self-efficacy, and intention. Grounded in the selective accessibility model (Mussweiler, 2003), the present research demonstrates that the effects of comparisons with more and less active others depend on the perceived similarity to that person. We found that participants evaluated their PA more favorably and felt more efficacious to be physically active when focusing on dissimilarities with a downward (Study 1 and 2) and similarities with an upward standard (Study 1).

Additionally, we showed that negative impacts of one’s initial similarity judgment can be partly compensated by considering the opposite of this judgment (Study 3). PA self-evaluation, self-efficacy, and intention improved when individuals considered the opposite of their initial impression that less active others are similar and more active others are dissimilar. By demonstrating the consider-the-opposite strategy’s applicability in the context of social comparison, our findings expand previous research on its use for correcting biased judgments of numeric values (Mussweiler et al., 2000) and food health claims (Chandon and Wansink, 2007). While positive (i.e. health-promoting) outcomes were observed in the downward initial similarity and upward initial dissimilarity consider-the-opposite conditions, there were no adverse effects in the upward initial similarity and downward initial dissimilarity consider-the-opposite conditions. It could be that participants put less effort into generating self-defeating information (e.g. evidence that one is performing poorly) to maintain a positive self-view. Exploring whether a protective mechanism like this exists, at which instances it occurs, and how it works present avenues for future research.

Accordingly, PA-promoting outcomes may be achieved by focusing on differences with less and similarities with more active others. Self-regulation tools (e.g. implementation intentions; “When I compare myself to a more active person, I will focus on similarities”; Gollwitzer, 1999) could help control similarity perceptions. Adverse effects of dissimilarity-focused comparisons with more and similarity-focused comparisons with less active others may be reduced by generating counterarguments (e.g. “Am I that different from this active person?”). Clinical applications utilizing social comparison to promote PA (e.g. treatment of obesity and noncommunicable diseases) should take individual factors, such as perceived similarity, into account and could benefit from applying the consider-the-opposite strategy.

The results also provide insights into the mediating roles of PA self-evaluation and self-efficacy in the relationship between comparison direction and PA intention. The impact of comparisons on beliefs about how active one is and how active one can be may thus explain *why* comparisons contribute to individuals’ intention to engage in PA. Specifically, the increase or decrease in PA intention may be attributable to evaluation and efficacy beliefs regarding the own PA. These results align with social-cognitive models stressing the importance of self-beliefs in health behavior change (Bandura, 2004).

The current findings should be interpreted in light of several limitations. First, the relationships between mediators and the dependent variable are correlational, preventing conclusions about whether PA self-evaluation and self-efficacy causally affect intention (Pirlott and MacKinnon, 2016). Second, given the PA intention-behavior discordance (Rhodes and de Bruijn, 2013), we cannot be certain about whether the observed changes in intention would cause behavior change. Research employing motion sensors or direct observation in laboratories (e.g. distance traveled on a treadmill) would offer valuable clarification.

## Conclusion

Collectively, our findings are promising from an individual-level perspective, as they suggest that effortful thought can be used to selectively increase the accessibility of similarity information and positively impact PA comparison effects. Efforts to focus on differences with less active and similarities with more active others may foster positive views of one’s abilities, which is essential to taking action.

## Supplemental Material

sj-docx-1-hpq-10.1177_13591053221086759 – Supplemental material for Perceived similarity determines social comparison effects of more and less physically active others Click here for additional data file. Supplemental material, sj-docx-1-hpq-10.1177_13591053221086759 for Perceived similarity determines social comparison effects of more and less physically active others by Iris Perey and Joerg Koenigstorfer in Journal of Health Psychology

## Notes

1 If “non-binary” or “prefer not to say” were chosen for gender, the standard description was randomly assigned.

2 See Supplemental Material for factor analysis and moderated mediation results using dissimilarity as moderator.

3 See Supplemental Material for moderated mediation analyses with difference scores reflecting outcome changes from T1 to T2.
