# Supplementary material for: Perceived similarity determines social comparison effects of more and less physically active others
Source: J Health Psychol. 2022 Apr 18;28(2):162–75. doi: 10.1177/13591053221086759 (PMC9936446; doi:10.1177/13591053221086759)
Supplement: sj-docx-1-hpq-10.1177_13591053221086759 – Supplemental material for Perceived similarity determines social comparison effects of more and less physically active others [file sj-docx-1-hpq-10.1177_13591053221086759.docx]

**Supplemental Material**

for:

Perceived Similarity Determines Social Comparison Effects

of More and Less Physically Active Others

Table of contents

[A. Social Comparison Task Descriptions 2](#_Toc79487988)

[B. Supplementary Analysis 1: Exploratory Principal Factor Analysis with Perceived Similarity Items for Study 1. 4](#_Toc79487989)

[C. Supplementary Analysis 2: Moderated Mediation Results with Perceived Dissimilarity as Moderator for Study 1. 5](#_Toc79487990)

[D. Supplementary Analysis 3: Moderated Mediation Results for Study 3. 7](#_Toc79487991)

# **Social Comparison Task Descriptions.**

***Female Upward Standard***

For as long as she can remember, Anna has engaged in some kind of sport. During her school time, she has been a member of the swim and volleyball team and participated in several other team sports. Until today, she has been swimming three times a week. In addition, Anna has a passion for running and strength training. She usually goes for a run two times a week and strengthens her muscles two times a week for 45 minutes each. In her daily life, Anna also looks for any possible opportunity to move. Although she does not live close to her office, she rides her bike to get there every morning, regardless of the weather conditions. This takes her about 30 minutes one way. Further, she takes the stairs and walks short to medium distances. Regular physical activity allows Anna to be strong and flexible at the same time. She can easily touch her toes when bending forward and she has no trouble carrying heavy groceries. She also has a good endurance, enabling her to climb the stairs to the third floor without arriving out of breath. Anna’s active lifestyle results in improved overall health and a longer life expectancy. Given her active and healthy lifestyle, she is among the privileged small group of people who have the very lowest risk for developing cardiovascular diseases, bone diseases, diabetes, obesity, and mental illnesses such as depression.

***Female Downward Standard***

For as long as she can remember, Anna has hated sports. She has never participated in any kind of sport during her time at school. She has tried out swimming, running, strength training as well as volleyball and several other team sports but doesn’t keep up doing any sports. During her leisure time, Anna prefers activities that require no or little body movement. If her friends ask her to join them on a hike in the mountains or to go swimming in summer, she usually refuses. In her daily life, Anna also tries to avoid any possible opportunity to move. Walking to her office would take her around 10 minutes. Although she lives close to the office, she takes public transport or the car to get there, every morning, because she finds walking extremely boring and avoids it as much as she can. Further, she tries to avoid stairs and takes the elevator or the escalator instead. Since Anna does not move a lot, she is weak and inflexible. She is not able to reach her toes when bending forward and has trouble carrying heavy groceries. She also has a bad endurance, which leaves her out of breath when climbing the stairs from the ground floor to the first floor. Anna’s inactive lifestyle results in decreased overall health and a shorter life expectancy. Given her inactive and unhealthy lifestyle, she is in the small group of people who have the very highest risk for developing cardiovascular diseases, bone diseases, diabetes, obesity, and mental illnesses such as depression.

***Male Upward Standard***

For as long as he can remember, John has engaged in some kind of sport. During his school time, he has been a member of the swim and volleyball team and participated in several other team sports. Until today, he has been swimming three times a week. In addition, John has a passion for running and strength training. He usually goes for a run two times a week and strengthens his muscles two times a week for 45 minutes each. In his daily life, John also looks for any possible opportunity to move. Although he does not live close to his office, he rides his bike to get there every morning, regardless of the weather conditions. This takes him about 30 minutes one way. Further, he takes the stairs and walks short to medium distances. Regular physical activity allows John to be strong and flexible at the same time. He can easily touch his toes when bending forward and he has no trouble carrying heavy groceries. He also has a good endurance, enabling him to climb the stairs to the third floor without arriving out of breath. John’s active lifestyle results in improved overall health and a longer life expectancy. Given his active and healthy lifestyle, he is among the privileged small group of people who have the very lowest risk for developing cardiovascular diseases, bone diseases, diabetes, obesity, and mental illnesses such as depression.

***Male Downward Standard***

For as long as he can remember, John has hated sports. He has never participated in any kind of sport during his time at school. He has tried out swimming, running, strength training as well as volleyball and several other team sports but doesn’t keep up doing any sports. During his leisure time, John prefers activities that require no or little body movement. If his friends ask him to join them on a hike in the mountains or to go swimming in summer, he usually refuses. In his daily life, John also tries to avoid any possible opportunity to move. Walking to his office would take him around 10 minutes. Although he lives close to the office, he takes public transport or the car to get there every morning because he finds walking extremely boring and avoids it as much as he can. Further, he tries to avoid stairs and takes the elevator or the escalator instead. Since John does not move a lot, he is weak and inflexible. He is not able to reach his toes when bending forward and has trouble carrying heavy groceries. He also has a bad endurance, which leaves him out of breath when climbing the stairs from the ground floor to the first floor. John’s inactive lifestyle results in decreased overall health and a shorter life expectancy. Given his inactive and unhealthy lifestyle, he is in the small group of people who have the very highest risk for developing cardiovascular diseases, bone diseases, diabetes, obesity, and mental illnesses such as depression.

# **Supplementary Analysis 1: Exploratory Principal Factor Analysis with Perceived Similarity Items for Study 1.**

In line with empirical evidence indicating that similarity and dissimilarity present distinct aspects of a comparison (Arigo et al., 2015, 2020), an exploratory principal axis factor analysis using oblique rotation revealed a two-dimensional structure. On this basis, separate mean scores were calculated, with higher scores demonstrating greater perceived (dis)similarity.

Table B1.

*Perceived Similarity Item-Factor Loadings for Study 1.*

|  | Item-Factor Loadings | |
| --- | --- | --- |
| Items | Perceived Dissimilarity | Perceived Similarity |
| While comparing yourself to [*Name of comparison standard*], … |  |  |
|  |  |  |
| how much did you focus on differences between yourself and [*Name*]? | .93 |  |
| to what extent did you think about how you are different from [*Name*]? | .92 |  |
| how much did you reflect on ways in which you and [*Name*] are different from each other? | .98 |  |
| to what extent did you pay attention to differences between yourself and [*Name*]? | .87 |  |
|  |  |  |
| how much did you focus on similarities between yourself and [*Name*]? |  | .95 |
| to what extent did you think about how you are similar to [*Name*]? |  | .96 |
| how much did you reflect on ways in which you and [*Name*] are similar to each other? |  | .91 |
| to what extent did you pay attention to similarities between yourself and [*Name*]? |  | .97 |
|  |  |  |
| Eigenvalues | 6.19 | 1.08 |
| Variance Explained (%) | 77.42 | 13.47 |

*Note*. *N* = 240. The response scale ranged from 1 = *not at all* to 5 = *most of the time*. Exploratory principal axis factor analysis results were obtained using oblique rotation. Item factor loadings are based on the pattern matrix. The factor solution was adequate for this data (Kaiser-Meyer-Olkin = .93; Barlette’s test of sphericity: χ^2^(28) = 2592.00, *p* < .001.

# **Supplementary Analysis 2: Moderated Mediation Results with Perceived Dissimilarity as Moderator for Study 1.**

We estimated the same moderated mediation model described under Study 1 but utilized perceived *dissimilarity* as moderator. To identify the nature of the potential interaction, conditional effects at low (mean -1 *SD*) and high (mean + 0.95 *SD*s^[[1]](#footnote-1)^) perceived dissimilarity were inspected. Results revealed significant effects of Comparison Direction × Perceived Similarity on PA self-evaluation, *B* = 1.14, *SE* = .11, *p* < .001, 95% CI [0.93; 1.34], and self-efficacy, *B* = 0.98, *SE* = .11, *p* < .001, 95% CI [0.75; 1.20] (see Figure C1 A). The conditional effect of comparison direction on PA self-evaluation was negative for low perceived dissimilarity, *B* = -0.68, *SE* = .15, *p* < .001, 95% CI [-0.97; -0.38], but positive for high perceived dissimilarity, *B* = 1.53, *SE* = .14, *p* < .001, 95% CI [1.25; 1.82] (see Table C1; Figure C1 B). The conditional effect of comparison direction on PA self-efficacy was negative for low perceived dissimilarity, *B* = -0.74, *SE* = .16, *p* < .001, 95% CI [-1.06; -0.42], but positive for high perceived dissimilarity, *B* = 1.16, *SE* = .16, *p* < .001, 95% CI [0.86; 1.47].

Congruent with our hypotheses, tests of conditional indirect effects suggested that the relationship between comparison direction and PA intention was mediated by PA self-evaluation at low, *B* = -0.34, *SE* = .10, 95% CI [-0.54; -0.15], and high perceived dissimilarity, *B* = 0.76, *SE* = .13, 95% CI [0.51; 1.03]. Likewise, the relationship between comparison direction and PA intention was mediated by PA self-efficacy at and low, *B* = -0.25, *SE* = .10, 95% CI [-0.47; -0.09], and high perceived dissimilarity, *B* = 0.40, *SE* = .10, 95% CI [0.21; 0.61]. Further, results revealed significant moderated mediation indices for PA self-evaluation, *B* = .56, *SE* = .10, 95% CI [0.38; 0.77], and self-efficacy, *B* = 0.33, *SE* = .10, 95% CI [0.17; 0.53].

Hence, the effects on PA self-evaluation and PA self-efficacy did not differ depending on whether perceived similarity or perceived dissimilarity was utilized as moderator in the model. This suggests that the conceptual difference between perceived similarity and dissimilarity, as presumed by Arigo et al. (2015, 2020), may be of minor importance in the present context.


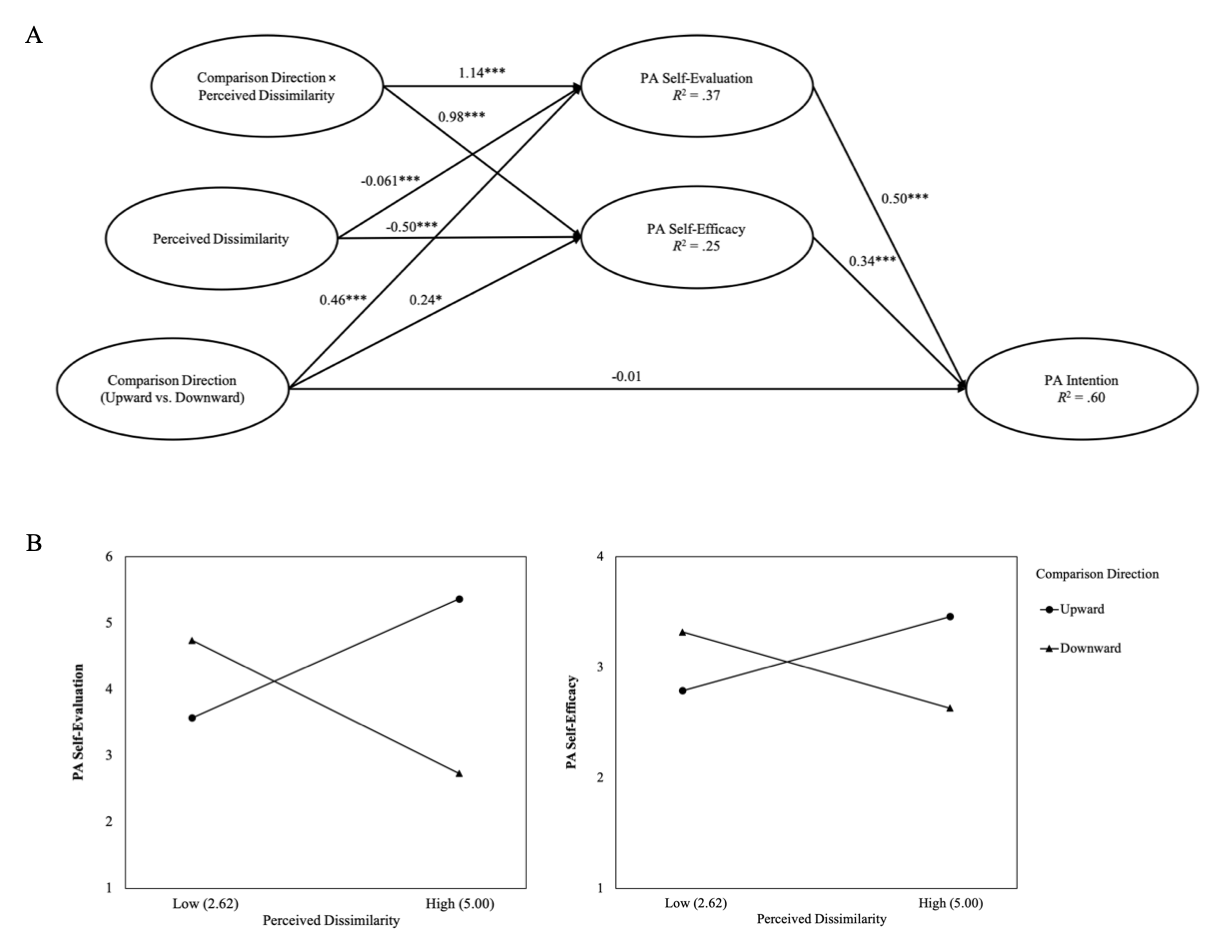


Figure C1. *Moderated Mediation Results for Study 1 with Perceived Dissimilarity as Moderator (A) and Conditional Effects of Comparison Direction on PA Self-Evaluation and Self-Efficacy at Low (Mean – 1 SD) and High (Mean + 0.95 SDs) Perceived Dissimilarity (B).
Note.* Mean + 0.95 *SD*s equals the highest possible score on the perceived dissimilarity response scale; PA = physical activity; response scales ranged from 1 = *not at all* to 7 = *very much* for PA self-evaluation, from 1 = *not at all* true to 4 = *always true* for PA self-efficacy, and from 1 = *not at all* to 5 = *most of the time* for perceived dissimilarity; * *p* < .05, *** *p* < .001 (two-tailed); coefficients are unstandardized.

Table C1.
*Means and Standard Deviations of PA Self-Evaluation and Self-Efficacy per Condition for Moderated Mediation Results with Perceived Dissimilarity as Moderator in Study 1.*

| Measures |  | PA Self-Evaluation | PA Self-Efficacy |
| --- | --- | --- | --- |
|  | *n* | *M(SD)* | *M(SD)* |
| Upward low Dissimilarity | 120 | 4.76(1.81) | 3.33(0.82) |
| Downward low Dissimilarity | 120 | 3.58(2.21) | 2.80(1.01) |
| Upward high Dissimilarity | 120 | 2.69(2.07) | 2.62(0.94) |
| Downward high Dissimilarity | 120 | 5.36(1.81) | 3.47(0.82) |

*Note*. PA = physical activity; low = mean – 1 *SD*; high = mean + 0.95 *SD*s (equaling the highest possible score on the perceived dissimilarity response scale). Response scales ranged from 1 = *not at all* to 7 = *very much* for PA self-evaluation and from 1 = *not at all true* to 4 = *always true* for PA self-efficacy.

# **Supplementary Analysis 3: Moderated Mediation Results for Study 3.**

We tested the proposed moderated mediation model with difference score variables reflecting the change in PA self-evaluation, self-efficacy, and intention from T1 to T2. The effects of Social Comparison × Consider-The-Opposite on PA self-evaluation and self-efficacy change were significant (*p*s < .001) as were the conditional effects of comparison direction on PA self-evaluation and self-efficacy change for both the initial similarity and the initial dissimilarity consider-the-opposite conditions (*p*s < .01). Conditional indirect effects and the index of moderated mediation were significant for PA self-efficacy change but non-significant for PA self-evaluation change. Further, change in self-efficacy, but not self-evaluation, were positively related to changes in intention.

Table D1.

*Moderated Mediation Results with Conditional and Conditional Indirect Effects for Consider-The-Opposite for Study 3.*

|  | Mediator:  PA Self-Evaluation Change | | Mediator:  PA Self-Efficacy Change | |
| --- | --- | --- | --- | --- |
| Moderator:  Consider-The-Opposite | *B*(*SE*) | 95 % CI | *B*(*SE*) | 95 % CI |
| Conditional Effects |  |  |  |  |
| Initial Similarity  Consider-The-Opposite | 0.62(.20)^**^ | 0.22; 1.02 | 0.54(.21)^**^ | 0.14; 0.95 |
| Initial Dissimilarity Consider-The-Opposite | -0.78(.15)^***^ | -1.07; -0.48 | -0.77(.15)^***^ | -1.06; -0.47 |
| Conditional Indirect Effects |  |  |  |  |
| Initial Similarity  Consider-The-Opposite | 0.03(.06) | -0.07; 0.17 | 0.18(.09) | 0.03; 0.38 |
| Initial Dissimilarity Consider-The-Opposite | -0.04(.07) | -0.19; 0.10 | -0.25(.12) | -0.50; -0.05 |
| Index of Moderated Mediation | -0.07(.13) | -0.35; 0.16 | -0.43(.19) | -0.83; -0.08 |

Note. *N* = 244; Comparison standard was used as predictor and PA intention change as outcome; PA = physical activity; **p < .01, ***p < .001 (two-tailed); CIs indicate significance for conditional indirect effects and indices of moderated mediation; coefficients are unstandardized.


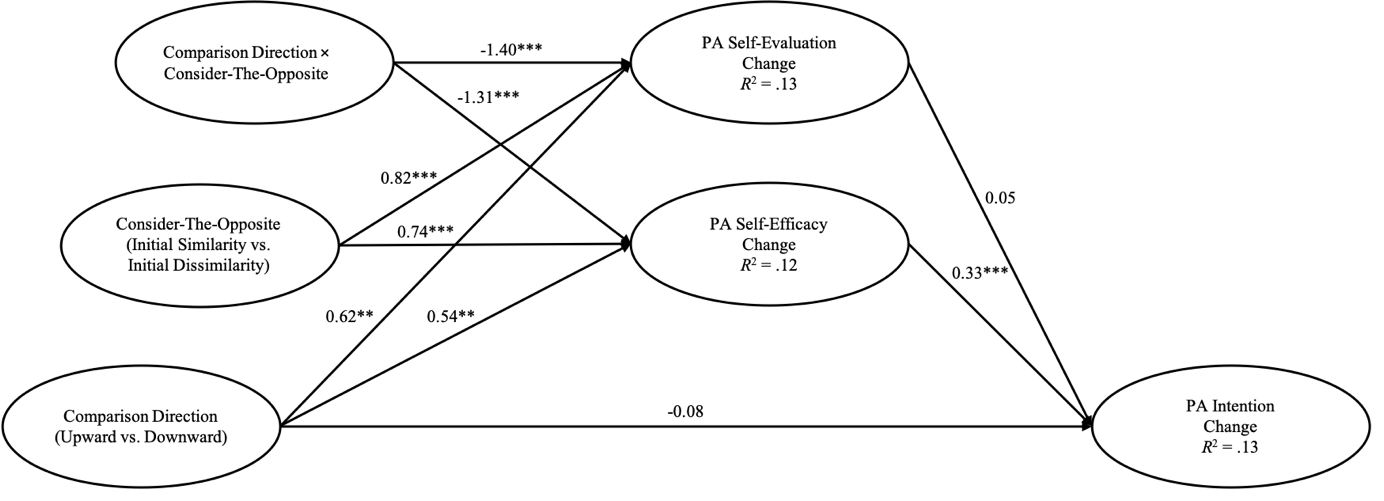


Figure D2. *Moderated Mediation Results for Study 3.*

*Note.* PA = physical activity; **p < .01, *** *p* < .001 (two-tailed); coefficients are unstandardized.

**References**

Arigo, D., Mogle, J. A., Brown, M. M., Pasko, K., Travers, L., Sweeder, L., & Smyth, J. M. (2020). Methods to assess social comparison processes within persons in daily life: A scoping review. *Frontiers in Psychology, 10*, 2909. <https://doi.org/10.3389/fpsyg.2019.02909>

Arigo, D., Smyth, J. M., & Suls, J. M. (2015). Perceptions of similarity and response to selected comparison targets in type 2 diabetes. *Psychology & Health, 30*(10), 1206-1220. <https://doi.org/10.1080/08870446.2015.1040018>

1. Mean + 0.95 *SD*s equals the highest possible score on the perceived dissimilarity response scale. [↑](#footnote-ref-1)
